# Prospective study of the Iliac Bicrest Pubic Angle through the 3D reconstruction of the bone pelvis and the correlation with giant incisional hernia

**DOI:** 10.1590/0100-6991e-20223130-en

**Published:** 2022-06-06

**Authors:** CARLOS JOSÉ LAZZARINI MENDES, RODRIGO ALTENFELDER SILVA, MARCELO DE CASTRO JORGE RACY, VINICIUS CASTRO DE REZENDE FIOROT, SERGIO ROLL, ADHEMAR MONTEIRO PACHECO

**Affiliations:** 1 - Faculdade de Ciências Médicas da Santa Casa de São Paulo, Departamento de Cirurgia - São Paulo - SP - Brasil; 2 - Irmandade da Santa Casa de Misericórdia de São Paulo, Departamento de Radiologia - São Paulo - SP - Brasil

**Keywords:** Hernia, Abdominal Wall, Bone and Bones, Anatomy, Hernia Incisional, Anatomia, Ossos, Cirurgia, Parede Abdominal

## Abstract

**Objective:**

to describe and measure the Bicrista Iliaca Pubo Angle (APBCI) as a new anthropometric parameter. Correlate the measurement with patients with giant incisional hernia (HIG), in the midline of the anterior abdominal wall (AAW).

**Methods:**

measurement of APBCI, through 3D reconstruction from computed tomography (CT). Measurements performed by two observers, R and C, in 246 women and 60 men, normal adults, in order to obtain the APBCI measurement and its correlation in patients with HIG of the AAW.

**Results:**

after sample calculations, the measurement of APBCI in men: 92.5+6.3º to 93.8+6.7º; in women: 90+6.7° to 94.3+6.8° [p-value 0.337(R)/0.628(C)]. The mean age was 57.9+15.9 years (22 to 91 years). Female gender 57+15.7 years (22 to 91 years) and male 61.7+16.5 years (23 to 89 years) p=0.067. As for the distribution of the ranges from 5 to 5 degrees, there is no difference in the distribution of the angle [p-value 0.455(R)/0.672(C)]. The correlation between age and angle showed that the higher the age, the higher the APBCI. There was no variability between angle measurements: 0.97 (95% CI 0.97; 0.98). In men with HIG, the average is between 108.3+5.37º (102.92º to 113.67º), and in women, 107.8+6.64 (101.16º to 114.44º).

**Conclusion:**

the study allowed us to conclude that HIG is not just an isolated AAW defect. Determines skeletal changes, as the APBCI is influenced by the distance of the iliac crests.

## RESUMO

**Objetivo:**

descrever e medir o Ângulo Pubo Bicrista Iliaca (APBCI) como novo parâmetro antropométrico. Correlacionar a medida com portadores de hérnia incisional gigante (HIG), da linha média da parede anterior do abdome (PAA).

**Métodos:**

medida do APBCI, através de reconstrução 3D a partir de tomografia computadorizada (TC). Realização de medidas por dois observadores, R e C, em 246 mulheres e 60 homens, adultos normais, afim de obter a medida do APBCI e sua correlação em portadores de HIG da PAA.

**Resultados:**

após cálculos de amostra, a medida do APBCI nos homens: 92,5+6,3º a 93,8+6,7º; nas mulheres: 90+6,7º a 94,3+6,8º [p-valor 0,337(R)/0,628(C)]. A média de idade foi de 57,9+15,9 anos (22 a 91 anos). Gênero feminino 57+15,7 anos (22 a 91 anos) e o masculino 61,7+16,5 anos (23 a 89 anos) p=0,067. Quanto à distribuição das faixas de 5 em 5 graus, inexiste diferença na distribuição do ângulo [p-valor 0,455(R)/0,672(C)]. A correlação idade e o ângulo demonstrou que quanto maior a idade, maior o APBCI. Não houve variabilidade entre as medidas do ângulo: 0,97 (IC95% 0,97; 0,98). Nos homens com HIG, a média está entre 108,3+5,37º (102,92º a 113,67º), e nas mulheres 107,8+6,64 (101,16º a 114,44º).

**Conclusão:**

o estudo permitiu concluir que a HIG não é apenas um defeito da PAA isolado. Determina alterações esqueléticas, na medida que o APBCI sofre a influência quanto ao afastamento das cristas ilíacas.

## INTRODUCTION

Primary or recurrent incisional hernias (IH) of the anterolateral abdominal wall (AAW), especially those of the midline and giant (GIH), whose transverse diameter of the hernia ring is ≥10cm, have been a challenge to surgeon, both in their correction and in the interpretation of their pathophysiological consequences^1^ ^-^ ^3^.

More than 2,000,000 laparotomies are performed in the United States of America, of which 150,000 require reoperations due to incisional hernias^4^ ^,^ ^5^.

Despite the efforts, incisional hernia is a frequent complication, occurring in 11% to 50% of patients undergoing laparotomies^6^ ^-^ ^8^. The recurrence rates of incisional hernioplasties can reach 19% to 30% of cases^9^.

According to Poulose et al., in 2012, the surgical correction of ventral hernia increases in incidence and costs. A 1% reduction in relapsed cases would save 32 million dollars a year^10^.

Surgical corrections of giant hernias behave as a major risk factor for poor evolution. A prospective study involving 3,258 incisional hernioplasties revealed a rate of 13.3% of hospital readmissions, 2.2% of reoperations, and 0.5% mortality^11^.

There are several risk factors for hernia recurrence. However, we did not observe studies that indicate whether the bone morphology of the pelvis would be involved in the pathophysiological complications of GIH and its influence in the AAW reconstruction^12^ ^,^ ^13^.

Regarding the classification of IH, there were numerous proposals, few being widely used, and in these, the bone component of insertion of the abdominal wall musculature was not considered^14^ ^-^ ^17^.

In 2009, the European Hernia Society proposed a classification for incisional hernias, assigning parameters to the topography of the anterolateral abdominal wall (AAW) in terms of area and defect size^18^.

AAW hernioplasties can be performed through primary sutures, with or without the interposition of prostheses, through the separation of the myoaponeurotic planes, aiming at their sliding towards the midline^19^.

As for AAW stratigraphy, it is necessary to consider that both the external oblique muscle (EOM), the internal oblique muscle (IOM), and transverse abdominis muscle (TAM) have an intimate relationship with the bone pelvis, that is, the iliac crests. The EOM is inserted in the anterior half of the iliac crest, the IOM’s origin is in the two anterior thirds of the iliac crest, and the TAM’s origin is the iliac crest^20^ ^,^ ^21^.

Aware of the eminently anatomical question, Radojevic, in 1958 to 1962, critically analyzed the pathophysiology of inguinal hernias, describing its predisposition by measuring the pelvic angle (Figure 1A)^22^ ^,^ ^23^.

**Figure 1A:**
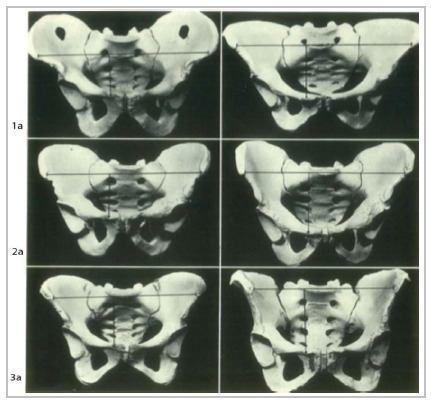
Radojevic Angle.

Stoppa, in 2002, highlighted the work of Barbin, in 1976, and Ami, in 1964, who also described the pelvic angle between the lines from the height of the pubic tubercle (PT) to the bicrest line (BCL) with the PT line to the anterosuperior iliac spine (Figure 1B)^24^.

**Figure 1B:**
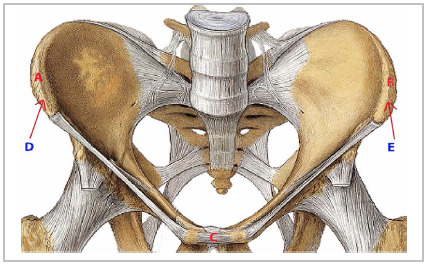
Pelvis: IBCPA landmarks.

*A: most lateral point of the right iliac crest (intermediate line). B: most lateral point of the left iliac crest (intermediate line). C: pubic symphysis. D: intermediate line of the right iliac crest. E: intermediate line of the left iliac crest.*

Young et al., in 1940, published a radiological study comparing female and male pelvises, demonstrating that the transverse diameter in men is smaller by an average of 16mm^25^ ^,^ ^26^.

Alberge et al., in 1985, studied pelvimetry through digital radiology generated by computed tomography, measuring the diameter of the pelvis^27^.

Lenhard et al., in 2009, studied pelvimetry with 3D reconstruction of computed tomography, concluding that it is easy and quick to assess, with low interobserver variability^28^.

Kim et al., in 2012, evaluated the accuracy of measurements in 14 frozen pig knees, verifying less than 0.3mm difference between the manual measurement versus the one performed by the OSIRIX Software, concluding that there was a strong correlation with real measurements and excellent inter and intra-observer reproducibility^29^ ^-32^.

The technique of Component Separation was certainly a big step in incisional hernioplasty. However, it does not make use of the musculoskeletal topography. Based on the premises, combined with the fact that not infrequently during the surgical procedure we observe tension points in the reconstruction of the midline of the AAW, especially in the infraumbilical region, we set out to describe and measure an angle, which we called Iliac Bicrest Pubic Angle (IBCPA), not described in the literature, to verifying if the measurements in normal (without hernia) adult men and women is correlated with patients with giant incisional hernias.

## OBJECTIVES

### General

To describe and measure the Iliac Bicrest Pubic Angle by digital pelvimetry as a new anthropometric parameter in normal adults.

### Specific

To assess the correlation of the IBCPA of normal individuals with bearers of GIH of the Midline or Medial Zones of the anterior abdominal wall.

## METHODS

### Angle description

The IBCPA is formed by the most lateral points of the iliac crest, positioned in the intermediate line of the iliac bone, on the right and left (origin of the internal oblique and transverse abdominis muscles) and the upper border of the pubic symphysis, in a plane that passes through the right and left pubic tubercles (Figure 1B).

**Figure 2:**
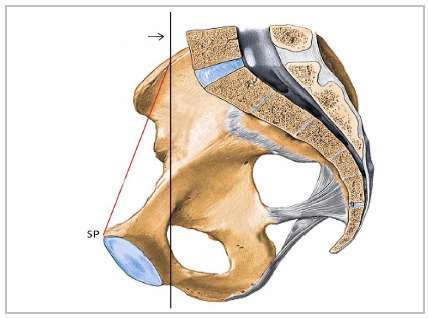
Right hemi-pelvis.

The IBCPA is inserted in an inverted base triangle. The base of the triangle is in a cranial position in relation to the angle, posterior to the pubic symphysis, taking the coronal plane as a reference (Figure 2).

*Red line: slope of the plane of the triangle formed to measure the IBCPA. PS: pubic symphysis. Black line: Coronal plane: Intermediate line of the right iliac crest. pubic symphysis: pubic symphysis.*

### Sample Calculation

The sample size for the IBCPA measurement was calculated with a significance level of 1% and an error of ±1º for each sex. From a pilot sample of ten women and ten men, the computed sample size was 245 women and 60 men (without hernia).

We also computed the sample to obtain measurements of the same angle in GIH bearers, resulting in 18 individuals (10 females and 8 males).

### Inclusion Criteria

- Men and women, adults over 21 years old.
- Normal pelvises.
- Images acquired with the patient in the horizontal supine position.

### Exclusion Criteria

- Abnormalities or anatomical variations that prevented measurements.
- Presence of bone prostheses or orthoses.
- Prior pelvic or hip surgery.
- Imaging artifacts that impaired bone measurements.

The Project was approved by the Ethics in Research Committee, opinion 862,177. Images were captured from computed tomography scans of the abdomen and pelvis at the Diagnostic Imaging Department, in DICOM format, 1.0mm thick and 1.0mm in increment, to perform the 3D reconstruction.

Two observers, one a general surgeon (C) and the other a radiologist (R), performed the measurements independently and blindly. Measurements were performed three times for each research individual, so that, for the 305 (245 female and 60 male) cases, 915 measurements were taken by each observer, being later averaged.

**Figure 3:**
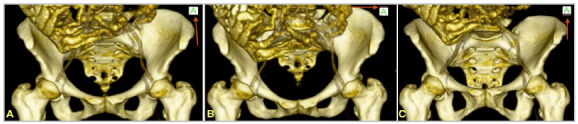
A. Pelvis, three-dimensional reconstruction 1. Red arrow shows Navigator Cube in anatomical position. B. Pelvis, three-dimensional reconstruction 2. Red arrow shows Navigator Cube in craniocaudal rotation position. Wrong position. C. three-dimensional reconstruction 3. Red arrow shows Navigator Cube in caudocranial rotation position. Wrong position.

**Figure 4:**
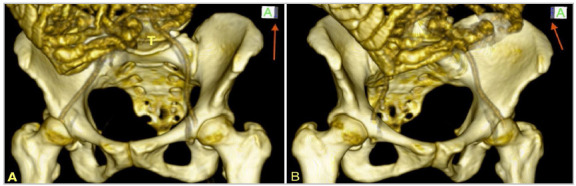
A. A. Pelvis, three-dimensional reconstruction. Red arrow shows Navigator Cube in right side rotation position. Wrong position. B. Pelvis, three-dimensional reconstruction. Red arrow shows Navigator Cube in left lateral rotation position. Wrong position.

The measurement was performed using the OSIRIX MD Software, from PIXEMEO. To perform the three-dimensional reconstruction, we used a Wacon Tablet Intuos CTL-480 Digitizing Table. After the 3D reconstruction of the pelvis, we used the 3D Position Navigator Cube of the Osirix MD Software, which provides six planes: Anterior (A), Posterior (P), Superior (S), Inferior (I), Right Side (R), and Left Side (L). We set the pelvises in an anatomic-virtual position, so that the horizontal lines of the Navigator Cube on its anterior face (Figures 3 and 4) remained as parallel as possible, avoiding measuring the pelvis in positions different from each other. 

**Figure 5:**
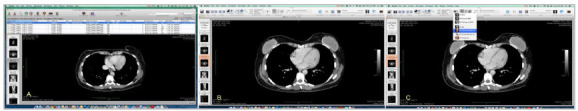
A. Image Import Screen - Osirix MD. B. Sequence selection screen: 1.0mm thick and 1.0mm increment - OSIRIX MD. C. Conversion screen for three-dimensional volume - “3D Volume Rendering” - OSIRIX MD .

**Figure 6:**
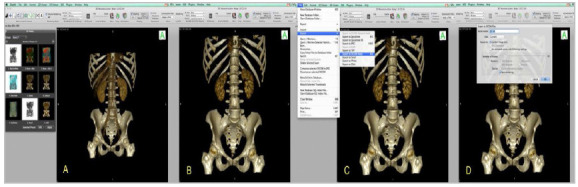
A. Bone modality preset conversion screen - “Group Bone CT: glossy” - OSIRIX MD. B. Screen of image selected and manipulated with three-dimensional rotation for anatomical position. A: cube demonstrating 3D anatomical position - OSIRIX MD. C. Screen of DICOM format image export in for angle measurement - software opening screen - OSIRIX MD. D. Screen of image export for angle measurement - software opening screen with high quality (“Best redering”) - OSIRIX MD .

**Figure 7:**
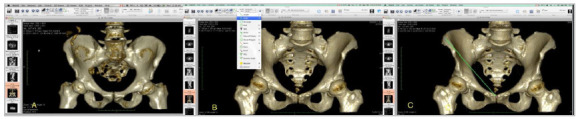
A. Screen of image exported for measurement of IBCPA - OSIRIX MD. B. “Angle” tool selection screen and first line drawing for angle measurement - OSIRIX MD. C. First IBCPA line drawing screen - OSIRIX MD .

**Figure 8:**
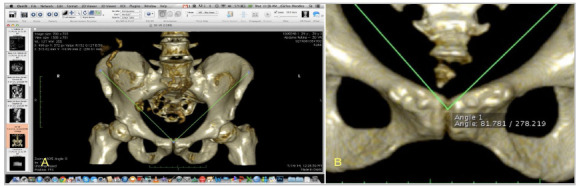
A. Second IBCPA line drawing screen - OSIRIX MD. B. Screen of drawn and measured IBCPA - OSIRIX MD.

We performed the following steps in OSIRIX MD:

1. Import of images with exam anonymization (Figure 5 A).
2. Sequence selection of 1.0mm thickness and 1.0mm increment (Figure 5B).
3. Conversion to three-dimensional volume - “3D Volume Rendering” (Figure 5C).
4. Conversion of Pre-selection bone modality - “Group Bone CT: glossy” (Figure 6A).
5. Selected image, worked with three-dimensional rotation for anatomical position, avoiding rotation of the pelvis that could impair the measurement of the IBCPA (Figure 6B).
6. Export of the image of item 5 in DICOM format for angle measurement on the opening page of the software (Figures 6C and 6D).
7. Image exported for angle measurement (Figure 7A).
8. Selection of the “Angle” tool (Figure 7B).
9. Drawing of first line to form the IBCPA (Figure 7C).
10. Drawing of second line to form the IBCPA (Figure 8A).
11. IBCPA formed, measured, and exported in JPEG (Joint Picture Experts Group) format for archiving (Figure 8B).

## RESULTS

We describe results through absolute and relative frequencies in the case of qualitative variables (sex), and median for the quantitative variables (age and angle value) and in the case of non-normal distribution, as verified by the Shapiro-Wilks test. We describe variables whose normality assumption was accepted through mean and standard deviation.

We compared categorical variables using the Fisher’s exact test. Continuous, non-normal variables were compared with the Mann-Whitney test. Normally distributed variables were compared using the Student’s t-test.

We performed the analysis of agreement between the three measurements performed by the surgeon, the radiologist, and between them using the Intraclass Correlation Coefficient (ICC). To analyze the measurements’ reliability, we used the Variability Coefficient on 12 randomly chosen individuals, three from each percentile, measuring the angle seven times, also randomly and blindly by the same observer. 

Analyzes were performed using the SPSS software, version 18.0 (SPSS Inc. Released 2009. PASW Statistics for Windows, Version 18.0. Chicago: SPSS Inc.).

### Descriptive and statistical analysis

The study was carried out with 305 individuals, 245 females and 60 males, and measurements were taken three times by each observer.

The mean age was 57.9 ± 15.9 years (22-91), 57 ± 15.7 years (22-91) for females and 61.7 ± 16.5 years (23-89) for males, p=0.067.

**Chart 1:**
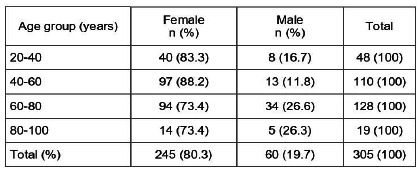
Distribution by age group.

We grouped the individuals in 20 year range. Most women and men were in the range between 60 and 80 years, 94/245 and 34/60 individuals, respectively (Chart 1).

The mean angle was 94.3º ± 6.8º (75.4º-117.1º) in women and 93.8º ± 6.7º (82.4º-111º) in men for the Radiologist measurements (MEAN-R), and 93º ± 6.7º (72.7º-116.1º) in women and 92.5º ± 6.3º (79.4º-109.2º) in men for the Surgeon measurements (MEAN-C). There was no difference regarding sex as to the MEAN-R and MEAN-C (p=0.337 and p=0.628) (Table 1).

**Table 1:** Descriptive analysis of ages and mean angles measured by the Radiologist and the Surgeon.

Variable	Group	Mean	Median	SD	Minimum	Maximum	p
Age	Female	57.0	57.0	15.7	22.0	91.0	0.067**
	Male	61.7	65.5	16.5	23.0	89.0	
MEAN-R	Female	94.3	94.6	6.8	75.4	117.1	0.337**
	Male	93.8	92.5	6.7	82.4	111.0	
MEAN-C	Female	93.0	93.0	6.7	72.7	116.7	0.628*
	Male	92.5	92.2	6.3	79.4	109.2	

*Student’s t-test / **Mann-Whitney test; SD: Standard Deviation.

We divided the angles in five degree ranges. The frequencies of the surgeon’s (MEAN-C) and radiologist’s (MEAN-R) averages were calculated, displaying no significant difference when sex was compared as a dependent variable.

Most male and female individuals had angles between 91º and 95º. However, for the MEAN-C in the 96º 100º range in women and the 91º-95º range in men for MEAN-R, the differences were not significant, p=0.672 and p=0.455, respectively (Tables 2 and 3).

**Table 2:** Distribution according to five-degree ranges and sex (Surgeon).

fx MEAN-C	Female n (%)	Male n (%)	Total	p
70º-75º	1 (100)	0 (0)	1 (100)	
76º-80º	3 (75.0)	1 (25)	4 (100)	
81º-85º	29 (85.3)	5 (14.7)	34 (100)	
86º-90º	47 (74.6)	16 (25.4)	63 (100)	
91º-95º	70 (76.9)	21 (32.1)	91 (100)	
96º-100º	62 (87.3)	9 (12.7)	71 (100)	
101º-105º	24 (82.8)	5 (17.2)	29 (100)	
106º-110º	8 (72.7)	3 (27.3)	11 (100)	
116º-120º	1 (100)	0 (0)	1 (100)	
	245 (80.3)	60 (19.7)	305 (100)	0.672

fx: frequency; MEAN-C: Surgeon’s measurements mean.

**Table 3:** Distribution according to five-degree ranges and sex (Radiologist).

fx MEAN-R	Female n (%)	Male n (%)	Total	p
76º-80º	2 (100)	0 (0)	2 (100)	
81º-8 5º	20 (80.0)	5 (5)	25 (100)	
86º-90º	42 (77.8)	12 (22.2)	54 (100)	
91º-95º	71 (75.5)	23 (24.5)	94 (100)	
96º-100º	64 (85.3)	11(14.7)	75 (100)	
101º-105º	33 (89.2)	4 (10.8)	37 (100)	
106º-110º	10 (76.9)	3 (23.1)	13 (100)	
111º-115º	2 (50)	2 (50)	4 (100)	
116º-120º	1 (100)	0 (0)	1 (100)	
	245 (80.3)	60 (19.7)	305 (100)	0.455

fx: frequency; MEAN-R: Radiologist’s measurements mean.

We verified the agreement between the Surgeon’s and the Radiologist’s measurements in the five-degree ranges. We observed agreement in 68.9% of the cases; in 28.2%, the Radiologist’s measurement was greater, and in only 3%, the Surgeon’s measurement was greater (Table 4).

**Table 4:** Analysis of agreement in percentage of measurement by angle range.

		Surgeon										Total
	Angle	70-75	76-80	81-85	86-90	91-95	96-100	101-105	106-110	111-115	116-120	
Radiologist	70-75	0	0	0	0	0	0	0	0	0	0	0
	76-80	1	1	0	0	0	0	0	0	0	0	2
	81-85	0	3	22	0	0	0	0	0	0	0	25
	86-90	0	0	12	38	3	1	0	0	0	0	54
	91-95	0	0	0	23	66	5	0	0	0	0	94
	96-100	0	0	0	2	22	51	0	0	0	0	75
	101-105	0	0	0	0	0	14	23	0	0	0	37
	106-110	0	0	0	0	0	0	5	8	0	0	13
	111-115	0	0	0	0	0	0	1	3	0	0	4
	116-120	0	0	0	0	0	0	0	0	0	1	1
	Total	1	4	34	63	91	71	29	11	0	1	305

We used the Spearman’s correlation test to verify if the correlation between age and pelvic angle was different from zero. All the correlations of the average age, both overall and by sex, were different from zero, positive, and weak to moderate, demonstrating that the higher the age, the higher the IBCPA (Table 5).

**Table 5:** Correlation test for age and angle.

Total	MEAN-R	MEAN-C	p
Average age	r=0.397	0.414	<0.001
Average female age	r=0.413	0.416	<0.001
Average male age	r=0.371	0.449	<0.001

We used a linear regression model to assess the amount of pelvic opening, in degrees, dependent on age.

The interpretation of these results showed that for each additional year of age, the IBCPA increases by 0.17 degrees. Below are the estimates of the fitted model (Table 6).

**Table 6:** Model estimates.

		Confidence Interval		
	Estimate	2.50%	97.50%	p
Age	0.17	0.15	0.19	<0.001
Male	-1.25	-2.99	0.48	0.16

### Intraclass Correlation Coefficient

We used the Intraclass Correlation Coefficient (ICC) to compare the MEAN-R and MEAN-C measurements. For the Surgeon’s measurements (MEAN-C) the ICC (intraobserver) was 0.998 (0.997- 0.998), while for the Radiologist’s (MEAN-R) the ICC was 0.998 (0.998-0.999). In the interobserver analysis the ICC was.982 (0.996-0.997), demonstrating strong and significant correlations.

### Variability Coefficient

We selected 12 individuals, three from each angle quartile, 72.17º-88.39º, 88.39º-92.79º, 92.79º 97.40º, and 97.40º-116.7º. The median of each interval was calculated so that, for each interval, we selected the three measures closest to the median. The Surgeon performed the measurements of each individual randomly and blindly seven times, unaware of the case to be measured (Table 7).

**Table 7:** Random measurements of the four angle quartiles and variability coefficient.

Range (°)	72.17-88.39			88.39-92.79			92.79-97.40			97.40-116.70		
Case no.	159	224	99	107	27	248	201	33	241	5	120	25
Measure 1 (°)	80.03	80.20	81.06	90.61	90.75	91.19	95.77	95.45	95.38	106.97	107.41	107.47
Measure 2 (°)	80.19	80.30	81.01	90.31	90.80	90.99	94.57	94.99	94.70	107.12	107.62	108.35
Measure 3 (°)	79.89	80.19	80.99	90.81	90.44	90.06	94.58	94.80	95.29	106.45	107.72	108.43
Measure 4 (°)	80.04	80.23	81.02	90.58	90.66	90.75	94.97	95.08	95.12	106.85	107.58	108.08
Measure 5 (°)	80.31	80.07	80.5	90.53	90.78	90.91	95	95.29	95.19	106.73	107.62	108.54
Measure 6 (°)	79.99	79.88	80.65	90.22	90.89	90.88	94.76	95.46	94.93	106.82	107.55	108.43
Measure 7 (°)	80	79.91	80.98	90.45	90.79	91.05	94.63	94.19	95.04	106.77	107.6	108.01
Var Coef (%)	0.2	0.1	0.2	0.2	0.1	0.3	0.4	0.4	0.2	0.1	0.8	0.3

Var Coef: variability coefficient; (°): angle.

When comparing the 18 adults with AAW GIH with normal ones (without hernia), the IBCPA measurement revealed the following values:

In men:

without hernia, the average of most angles was between 92.5º ± 6.3º and 93.8º ± 6.7º, that is, between 86.2º and 100.5º;

with hernia, the average was between 108.3º ± 5.37º (8 cases), that is, between 102.92º and 113.67º.

In women:

without hernia, the measure of most angles was between 90º ± 6.7º and 94.3º ± 6.8º, that is, between 83.3º and 101.1º;

with hernia, the average was between 94.64º ± 5.30º (18 cases) and 107.8º ± 6.64º (10 cases), that is, between 101.16º and 114.44º.

The results of the measurements revealed that in patients with hernia, the opening angle of the pelvis in men was 2.42º, and in women, 0.06º, greater than in normal individuals. 

## DISCUSSION

When we set out to describe and measure the IBCPA, we were moved by intraoperative observations that, after anterior separation of components, in some cases, it promoted squeezing of the infraumbilical region during the reconstruction of the midline of the abdomen, keeping tension. In the Component Separation technique proposed in 1990, Ramirez et al. already admitted the external oblique muscle (EOM) limited advance towards the midline^20^.

The pelvic opening could, in theory, interfere with the component separation technique, eventually behaving as a limiting factor for the adequate sliding and closing of the AAW. Our study demonstrated a higher IBCPA at the time of GIH.

Radojevic ‘s studies evaluated 250 male and 150 female pelvises and verified the angle formed between the line of the anterior-superior iliac spines and a line between the anterior- superior iliac spine and the pubic tubercle (PT). If the value exceeded 25º 30º, it would be suggestive of a large region for inguinal hernia formation, at that moment disregarding incisional hernias^22^ ^,^ ^23^.

Stoppa, in 2002, also studied pelvic angles between the height of the PT and the BCL, reporting that when lower than 60º-65º they would be associated with patients with inguinal hernia, in which the greater the height, the greater the incidence, though not considering major defects of the AAW^24^.

Despite the renowned studies by Turner in 1885, Thoms in 1937, Caldwell et. al in 1938, there were no parameters in these studies to explain the behavior of the bone pelvis at the time of GIH^19^. 

Surprisingly, the IBCPA measurements did not show differences, on average, between men and women, which is in line with the 18 types of pelvis described by Caldwell.

We expected women to have a greater average measurement; however, the study revealed greater variation in amplitude in degrees.

We also observed that the IBCPA is lower in men when comparing individuals of the same age and different genders. 

Considering aging, the IBCPA increases with age, so that we could hypothesize that the older the patient, the greater would be the difficulty of correcting the GIH in the Zones M3, M4, and M5 of the AAW, since the pelvis ultimately widens.

We adopted the premise that the horizontal dorsal decubitus during Computed Tomography maintains the neutrality of the pelvic positioning, which, in addition to promoting the evaluation of the hernia orifice, that is, the myoaponeurotic planes during hernias, at the same time allows the surgeon to measure the IBCPA through the method applied in this study.

The three-dimensional reconstruction of the pelvis and the measurements performed on it received standardization of position, of the vertices of the angle to be studied, so that they are reproducible, and were carried out as close as possible to the anatomical position. 

We understand that even though such measurements may suffer interference from the observer during the reconstruction and positioning, when “anatomical-virtual” parameters are set, like those described in our study, such errors can be avoided. These conditions, called ideal, do not always occur in clinical practice, causing the surgeon to face non-normal situations. However, we understand that we initially included in the study anatomically normal individuals for the measurement of the IBCPA. 

## CONCLUSION

### The study allowed us to conclude that:

GIH is not just an isolated AAW defect. It determines skeletal changes, as the IBCPA is influenced by the retraction of the iliac crests.

The presence of a giant incisional hernia is accompanied by a greater degree of opening of the pelvis, so that the midline reconstruction of the AAW is even more subjected to the tension forces of the increase in the IBCPA.

This offers the surgeon another parameter in the application and choice of reconstruction techniques of the midline of the anterolateral abdominal wall.
